# Comparison of A 1940 nm Thulium-Doped Fiber Laser and A 1470 nm Diode Laser for Cutting Efficacy and Hemostasis in A Pig Model of Spleen Surgery

**DOI:** 10.3390/ma13051167

**Published:** 2020-03-05

**Authors:** Bogusława Żywicka, Zbigniew Rybak, Maciej Janeczek, Albert Czerski, Jolanta Bujok, Maria Szymonowicz, Maciej Dobrzyński, Mariusz Korczyński, Jacek Świderski

**Affiliations:** 1 Department of Experimental Surgery and Biomaterial Research, Wroclaw Medical University, Bujwida 44, 50-368 Wroclaw, Poland; zbigniew.rybak@umed.wroc.pl (Z.R.); maria.szymonowicz@umed.wroc.pl (M.S.); 2 Department of Animal Physiology and Biostructure, Division of Anatomy, Wroclaw University of Environmental and Life Sciences, Kożuchowska 1, 51-631 Wroclaw, Poland; maciej.janeczek@upwr.edu.pl; 3 Department of Animal Physiology and Biostructure, Division of Animal Physiology, Wroclaw University of Environmental and Life Sciences, C.K. Norwida 31, 50-375 Wroclaw, Poland; albert.czerski@upwr.edu.pl (A.C.); jolanta.bujok@upwr.edu.pl (J.B.); 4 Department of Conservative Dentistry and Pedodontics, Wroclaw Medical University, Krakowska 26, 50-425 Wroclaw, Poland; maciej.dobrzynski@umed.wroc.pl; 5 Department of Environment Hygiene and Animal Welfare, Wroclaw University of Environmental and Life Sciences, Chełmońskiego 38c, 51-630 Wroclaw, Poland; mariusz.korczynski@upwr.edu.pl; 6 Institute of Optoelectronics, Military University of Technology, Kaliskiego 2, 00-908 Warsaw, Poland; jacek.swiderski@wat.edu.pl

**Keywords:** partial splenectomy, thulium-doped fiber laser (1940 nm), diode laser (1470 nm), pig model of spleen surgery, thermal damage zone, hemostasis

## Abstract

Partial and total splenectomies are associated with a high risk of substantial blood loss. Lasers operating at wavelengths strongly absorbed by water have the potential to improve hemostasis and cut while providing a narrow zone of thermal damage. The aim of this study is to compare a thulium-doped fiber laser (TDFL) emitting a wavelength of 1940 nm and a diode laser (DL) operating at 1470 nm for spleen surgery in a pig model. A partial splenectomy and spleen incisions were made in 12 animals using the two laser devices. The hemostasis was evaluated visually during surgeries. Post-mortem and histopathological evaluations were done on days 0, 7, and 14 following surgery. Neither TDFL nor DL caused bleeding on day 0 or delayed bleeding. On day 14, pale streaks at the site of incision were slightly wider after cutting with DL than with TDFL. Histological analysis revealed a carbonized zone with exudation and a deeper zone of thermal tissue damage on day 0. The width of the thermal changes was 655.26 ± 107.70 μm for TDFL and 1413.37 ± 111.85 μm for DL. On day 7, a proliferation of fibroblasts and splenocytes was visible, as well as a formation of multinucleated giant cells adjacent to the residues of carbonization. The zone of thermal damage was broader for DL (1157.5 ± 262.77 μm) than for TDFL (682.22 ± 116.58 μm). On day 14, cutting sites were filled with connective and granulation tissues with the residues of carbonization. The zone of thermal damage was narrower for TDFL (761.65 ± 34.3 μm) than for DL (1609.82 ± 202.22 μm). Thus, both lasers are efficient in spleen surgery, providing good hemostasis. However, TDFL produces a narrower zone of thermal damage, which suggests its better efficiency for spleen surgery, especially when performing more precise procedures.

## 1. Introduction

A significant percentage of spleen surgeries are performed to stop the hemorrhage caused by a traumatic rupture of the organ. To date, total splenectomy has been the method of choice for the treatment of severe splenic injuries. Other indications for surgical spleen removal include acquired hemolytic anemia, hypersplenism, autoimmune thrombocytopenia, myeloproliferative disorders, spherocytosis, spleen abscesses, cysts, tumors, and portal hypertension [1,2,3]. 

However, partial splenectomy is indicated in children and in adults with specific disorders. Leaving 25% of the organ reduces hemolysis while maintaining the phagocytic function of the spleen [4,5]. In recent years, the principles of treatment for spleen disorders have changed significantly. At present, systemic diseases are an absolute indication for splenectomy, whereas isolated spleen diseases are managed by partial resection. Moreover, organ-sparing surgery is recommended for traumatic spleen injury [6].

In clinical practice, tissue adhesives, stapling, coagulation, and photocoagulation are used for the surgical supply of spleen trauma [1,2,7]. A comparative analysis of laser technology versus scalpel cutting and suturing on surgical outcomes in traumatic spleen injury showed greater efficiency of laser surgery, which enabled spleen preservation in 63% of patients [8]. Moreover, laser coagulation was effective in stopping parenchymal bleeding and thus significantly reduced procedure duration and resulted in better organ sparing [9,10]. However, more recent reports on the use of lasers in spleen surgery are few. Moreover, there are no studies on the application of thulium-doped fiber laser for partial spleen resection.

Furthermore, current data on the cellular and tissue mechanisms of parenchymal scar formation suggest that laser surgical devices may minimize destructive processes, and therefore they are very promising in parenchymal organ surgery. However, optimal power parameters and a light wavelength at which the laser operates have to be determined for each parenchymal tissue to provide reliable and rapid wound healing and minimal thermal destruction of the surrounding tissues [11]. 

The wavelength of light emitted by a laser is an important parameter in terms of absorption by the water. Radiation at a wavelength of 1940 nm is effectively absorbed by water and does not penetrate deep into the tissue, which translates into higher ablation efficiency and greater precision of the procedure [12]. Although similar hemostasis in the parenchymal organs can be achieved with lower wavelengths of laser light, the degree of surrounding tissue destruction is not known [13]. Radiation at a wavelength of 1470 nm is absorbed by cellular water 40 and 160 times better than at the wavelengths of 980 nm and 1064 nm, respectively. This high absorption of a 1470 nm laser wavelength by water restricts tissue penetration and limits beam interaction with surrounding tissues.

Lasers operating at a wavelength of 1470 nm and providing an output power up to 20 W have proven to be useful in selected phlebological procedures, as well as in the surgical treatment of benign prostatic hyperplasia, and the evaluation of their usefulness, safety, and efficacy is still the subject of numerous studies [14,15]. 

A fiber laser with an active medium in the form of a thulium-doped (Tm^3+^) optical fiber (TDFL) working at a wavelength of 1940 nm shows nearly 1000 times greater absorption by water when compared with lasers emitting light at 1064 nm (Nd: YAG laser). This feature enables precise tissue ablation with a small margin of coagulation, while laser radiation of a 1064 nm wavelength penetrates deeper into tissue with less controlled coagulation effects [16,17,18]. 

Lasers operating at wavelengths of 1470 nm and 1940 nm seem to be the most prospective ones for soft-tissue surgery. The former are typically high-power semiconductor lasers delivering multimode radiation. Those used for medical applications are usually equipped with fiber pigtails with diameters ranging from 50 to 600 μm and typical numerical apertures (NA) of 0.22. Fiber lasers are classified as solid-state lasers, and their construction is much more complicated compared to diode lasers. They comprise an amplifying medium in the form of a rare earth-doped optical fiber and a resonator formed by two dielectric mirrors or two fiber Bragg gratings. For operation, they need optical pumping, which is realized by applying diode lasers. Compared with diode lasers, they provide single-mode output with a perfect beam quality, as the beam is usually diffraction-limited. Furthermore, the output beam is generated by a fiber with a core diameter of 25 μm or smaller and an NA below 0.1, which predestines these lasers for coupling with fiber medical probes with small diameters.

Comparative studies on the biological effects of both 1470 nm and 1940 nm surgical lasers are limited and focus on phlebological procedures or parenchymal organs, including nephrotomy, but in ex vivo conditions as an intermediate element for different research purposes [19,20].

The most frequently mentioned lasers in partial splenectomy operations and intraoperative hemostasis of the spleen are CO_2_ and Nd: YAG lasers [8,9,21]. The literature related to the use of lasers operating at 1470 nm and 1940 nm wavelengths for partial or total splenectomy is very scarce, and therefore, our study fills the existing gap in this area.

For many other surgical procedures, an optimal laser has not yet been developed [12,22]. Nowadays, surgical laser devices simultaneously producing good hemostasis and limited tissue penetration are sought for minimally invasive endoscopic and robotic surgery. They will reduce the procedure duration and will prevent unexpected intraoperative complications. Concurrently, the minimized tissue carbonization will alleviate the inflammatory response and accelerate the regeneration process. In response to the increasing demand in medicine, we have developed two high-power surgical lasers emitting light at the wavelengths of 1470 nm and 1940 nm.

The aim of our study was to compare a 1940 nm TDFL and a 1470 nm diode laser (DL) for spleen surgery in a pig model by analyzing the effectiveness of hemostasis and cutting, as well as the breadth of the thermal damage zone.

## 2. Materials and Methods

### 2.1. Lasers

The experiment was designed to test two surgical lasers producing high output powers and operating either at 1470 nm (DL) or 1940 nm (TDFL) for soft tissue surgery. The devices were developed by Metrum Cryoflex Sp. z o. o. (Blizne Łaszczyńskiego, Poland) in cooperation with the Military University of Technology.

The DL was a multimodal semiconductor laser generating continuous wave (CW) radiation of up to 100 W at a wavelength of 1470 nm. The method of power supply and laser control enabled the device to operate not only in a standard CW mode, but also in a quasi-continuous mode (QCW), in which it could generate optical pulses with a duration of 200 ms or more. The optical beam was directly launched into the laser probe with a diameter of 400 μm.

The TDFL was a single-mode laser operating at a wavelength of 1940 nm and delivering an output power of up to 34.7 W. The laser was developed using a double-clad fiber with a core/clad diameter of 25/250 μm, doped with thulium ions (Tm^3+^) pumped at 793 nm wavelength. The output radiation was characterized by a very good beam quality (parameter M^2^ ~ 1.2), and therefore it could easily be inserted into the treatment probes with a diameter smaller than 50 μm [17]. The details of the laser can be found in [23].

Based on a series of preliminary intraoperative cuts, we experimentally determined the minimal power for each test laser for inducing hemostasis during cutting of the spleen. Our goal was to cut the spleen with hemostasis while minimizing the destruction of surrounding tissues. We used the set powers throughout the experiment.

The laser probes were treated as a scalpel knife and pulled directly on the spleen’s surface with continuous motion at a constant average speed of 3.34 ± 0.38 mm/s.

Performing spleen surgery, both lasers worked in CW mode. The power of the Tm-doped fiber laser was 21 ± 1 W and the power of the diode laser was 50 ± 1 W. The power was measured at the end of the laser probes. During operation, the lasers’ parameters were kept constant for each application of the same laser. During this experiment, the lasers did not work in a QCW. The most important parameters of the lasers are listed in Table 1.

### 2.2. Experimental Animals

All of the experimental procedures were approved by the II Local Ethical Committee in Wrocław (Approval No. 87/2012) and performed in accordance with the directive of the EU (2010/63/EU). The experiments were conducted on 12 female pigs of Large Polish White breed aged 10 weeks and weighing approximately 30 kg on the day of surgery. The animals were purchased from a certified animal breeding farm (The National Research Institute of Animal Production, Experimental Station in Pawłowice, Poland), were free of halothane gene, and had a low body fat percentage. Before the experiment, each animal was acclimatized for 14 days in standard conditions of the vivarium. Six pigs, randomly selected before the laser surgery, were observed for 7 days following the procedure, and the remaining six animals for 14 days following the procedure.

### 2.3. Animal Preparation and Surgery

The animals were examined every day by a veterinarian. Then, 24 h before the planned surgery, feeding was discontinued, although the animals had unrestricted access to fresh drinking water. All surgical procedures were performed aseptically in an operating room by the same operators and anesthesiologists. A partial splenectomy and spleen incisions were made in the animals using both TDFL and DL. 

Pigs were pharmacologically sedated by an intramuscular (i.m.) injection of medetomidine (0.1 mg/kg body weight, Domitor®, Orion Pharma, Warsaw, Poland) and butorphanol (0.2 mg/kg body weight; Butomidor®, Richter Pharma AG, Wels, Austria). Afterward, peripheral venous catheters were inserted into the auricular veins of the animals and placed in dorsal recumbency. General anesthesia was induced with an intravenous (i.v.) bolus of propofol (4 mg/kg, Scanofol®, Scan Vet Sp. z o.o, Skiereszewo, Poland), followed by intratracheal intubation. General inhalation anesthesia was maintained with 1.5 vol % Isoflurane. Intraoperative analgesia was produced by a constant rate i.v. infusion of fentanyl at a dose of 500 μg/h (Polfa SA, Łódź, Poland). Each animal received 500 mL of Ringer solution i.v. during the procedure.

To access the spleen, a midline incision of the abdominal wall was done. Then, the organ was pulled out and placed on sterile gauze. Subsequently, a partial resection with DL or TDFL and an incision of the spleen with DL and then with TDFL at a distance of approximately 2 cm were performed. Both laser probes were held in direct contact with the surface of the spleen. The excised fragments of the spleen were harvested for histopathological evaluation immediately after cutting. Operators assessed the cutting efficiency and observed bleeding from the incision sites for 5 min during the procedures. The abdominal wall was closed with 3-0 absorbable sutures (Yavo, Ltd., Bełchatów, Poland), and the skin wound was closed with 2-0 non-absorbable sutures (Yavo, Sp. Z o.o., Bełchatów, Poland). Subsequently, the animals were administered amoxycillin i.m. (15 mg/kg body weight, Betamox LA®, ScanVet Sp. z o.o, Skiereszewo, Poland) and metamizole i.m. (30 mg/kg body weight, Biovetalgin®, Biowet Drwalew, Drwalew, Poland) and placed in boxes with free access to food and water. The animals from the first and the second groups were observed for 7 and 14 days after surgery, respectively. Following the laser surgery, pigs received metamizole i.m. (Biovetalgin, Biowet Drwalew, Drwalew, Poland) twice daily for three days.

### 2.4. Post-Mortem Examination

The animals were euthanized after 7 or 14 days post-surgery. Following adequate i.m. sedation, pigs were administered i.v. pentobarbitone (Morbital®, Biowet Puławy, Puławy, Poland) at a maximum dose of 120 mg/kg body weight until cardiac arrest. 

During the post-mortem examination of the spleen, particular attention was paid to the analysis of the cutting and resection sites. Afterward, spleen fragments were obtained for a histopathological evaluation of the extent of carbonization and cellular thermal changes.

### 2.5. Histopathological Examination

Spleen fragments were fixed for 72 h (at room temperature) in a 10% aqueous solution of formaldehyde (previously neutralized with calcium carbonate) in a phosphate buffer. After fixation, the spleen fragment was cut transversely to the line of laser cut into a series of tissue blocks with a side length of about 1 cm. The samples were then dehydrated in acetone (at 56 °C), cleared with xylene (at room temperature), and embedded in paraffin blocks, which were then cut into approximately 4-μm-thick sections (Leica 2025 rotational microtome, Leica Microsystems, Wetzlar, Germany). The specimens were stained with hematoxylin and eosin (HE; Sigma-Aldrich, Saint Louis, MO, USA) and then closed in a mounting medium (CV Mount Medium, Leica Biosystems GmbH, Nussloch, Germany). Tissue specimens were assessed under a light microscope (Olympus BX43, Olympus Corporation, Tokyo, Japan). The histological images were photographed and measurements were done using a cellSens standard analysis and acquisition imaging software (Version 1.6; 2010 Olympus Corporation, Tokyo, Japan). Approximately five to seven slides for each cut done with each laser for one animal were prepared. To assess the depth of the thermal changes, the average of the measurements for series of sections for each animal was taken into account.

### 2.6. Statistics

Data are expressed as the mean ± standard deviation for *n* animals. Data were analyzed by Student’s *t*-test using the Statistica for Windows Version 10.0 software package (StatSoft, Tulsa, OK, USA). Differences between means were considered significant when *p* ˂ 0.05.

## 3. Results

### 3.1. Intraoperative Visual Examination 

**Figure 1:**
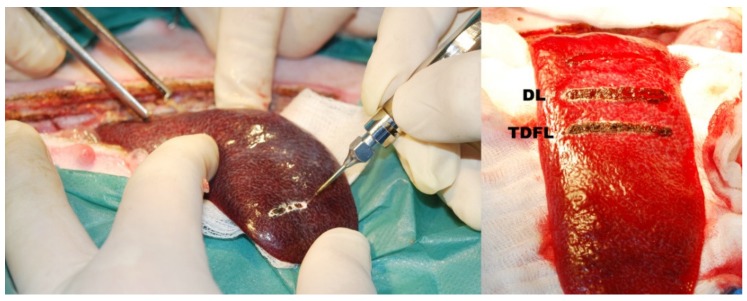
Pictures taken intraoperatively showing (**left**) the procedure of spleen incision with a laser probe, and (**right**) two incisions on the spleen made with a diode laser (DL) and thulium-doped fiber laser (TDFL).

High cutting efficiency of both types of lasers was demonstrated intraoperatively during spleen incisions and partial resection. Intraoperative observation of hemostasis either did not reveal or revealed only minimum transient bleeding from the incision and resection sites for both lasers. The incision sites after cutting with TDFL and DL had a similar appearance, forming carbonization streaks with an average width of 2 mm. However, TDFL tended to create narrower streaks of carbonization when compared with DL (Figure 1).

### 3.2. Clinical Outcome and A Post-Mortem Examination

**Figure 2:**
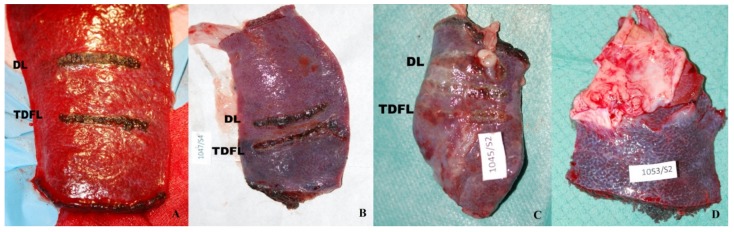
Macroscopical appearance of the spleen in pigs after incisions made with a thulium-doped fiber laser (TDFL) and a diode laser (DL). (**A**) On the day of surgery, (**B**) on day 7 post-surgery, (**C**) on day 14 post-surgery, (**D**) and the site of partial resection of the spleen with the diode laser (DL) 14 days post-surgery.

All animals were weaned from anesthesia without complications and survived until the planned euthanasia in a good clinical condition. During post-mortem examinations after 7 and 14 days following surgery, no extravasated blood or clots were found. On day 7 post-surgery, adhesions of the spleen with the mesentery, peritoneum, or stomach in the immediate vicinity of the site of cutting were present in the individual animals. Following excision, the spleen had a normal appearance in all pigs. Incision sites formed healing wounds with lighter streaks along the cutting line, slightly narrower for TDFL than for DL (Figure 2). The surrounding surface of the spleen was macroscopically normal. On day 14 post-surgery, in most animals, the spleen at the sites of cutting adhered to the intestine, peritoneum, or omentum. In some animals, the cutting sites were covered entirely. In the remaining animals, the spleen with a normal appearance and partially healed wounds or scars after cutting with TDFL and DL was visible (Figure 2). During the post-mortem examination, no pathological changes in the internal organs were found.

### 3.3. Histopathological Examination

Histopathological examination of the specimens collected intraoperatively and on day 7 post-surgery revealed superficial and deep zones of thermal damage induced by TDFL and DL. Fourteen days after the surgery, both zones merged. For all measurements during the experiment, the width of both the superficial and total thermal damage zones was significantly smaller for TDFL.

**Figure 3:**
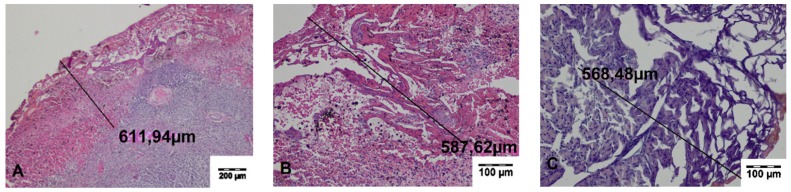
Histopathological sections of the spleen fragments collected intraoperatively, showing changes produced by the thulium-doped fiber laser (TDFL). Black lines indicate the width of the entire zone of thermal damage (hematoxylin and eosin (HE) staining). (**A**) A superficial zone of carbonization and a deeper zone of thermal changes are present in the spleen parenchyma (magnification × 40); (**B**,**C**) magnification × 100.

**Figure 4:**
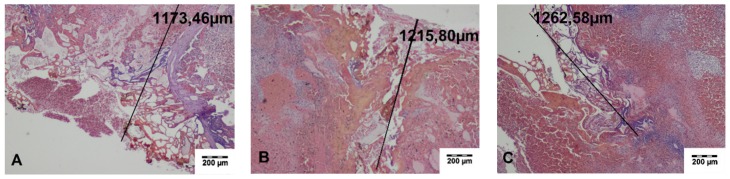
Histopathological sections of the spleen fragments collected intraoperatively at the site of cutting with the diode laser (DL). Black lines indicate the width of the entire zone of thermal damage (HE staining). (**A–C**) A quite wide superficial zone of the carbonized tissue and a deeper zone of thermal changes are visible (magnification × 40).

In the specimens collected during the surgery, a zone of carbonized tissue with exudation free of erythrocytes or with a small number of extravasated erythrocytes, as well as a zone of altered tissue, was visible. In the immediate vicinity of the incisions, superficial carbonized amorphous necrotic structures with an average width of 197.29 ± 35.99 μm for TDFL and 313.44 ± 20.89 μm for DL were found. The deeper zone was characterized by slightly shrunken cells with preserved cell nuclei, sharply demarcated from the surrounding normal tissue. The average width of both zones of thermal tissue damage was 655.26 ± 107.70 μm for TDFL and 1413.37 ± 111.85 μm for DL (Figure 3 and Figure 4, Table 2).

On day 7, at the site of cutting with both lasers, histopathological examination revealed residues of carbonized necrotic structures and exudation rich in inflammatory cells. Granulocytes, macrophages, lymphocytes, plasma cells, and forming giant cells were distinguished.

**Figure 5:**
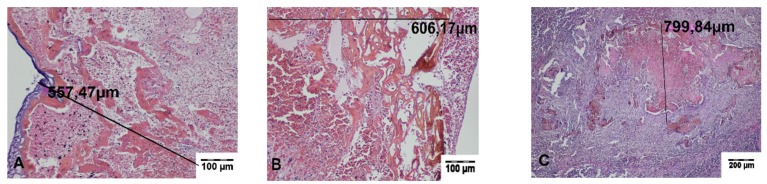
Histopathological sections of the spleen on day 7 following cutting with the thulium-doped fiber laser (TDFL). Black lines indicate the width of the entire zone of thermal damage (HE staining). (**A**,**B**) The superficial zone of the thermal damage (magnification × 100); (**C**) residues of the carbonized tissue surrounded by the parenchyma with visible processes of repair and regeneration (magnification × 40).

**Figure 6:**
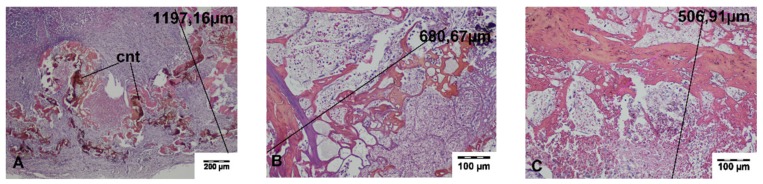
Histopathological sections of the spleen of the pig 7 days after cutting with the diode laser (DL) (HE staining). Black lines indicate the width of the entire zone of thermal damage. (**A**) Residues of the carbonized tissue surrounded by granulation tissue with proliferating fibroblasts and splenocytes in the deeper zone of thermal damage (magnification × 100); (**B**,**C**) superficial zone of thermally damaged tissue (magnification × 100). cnt, carbonized necrotic tissue.

The average width of these changes was 150.00 μm and 405.92 μm for TDFL and DL, respectively. The deeper zone with inflammatory granulation tissue, proliferating fibroblasts, and splenocytes was sharply demarcated from the normal spleen tissue. The average total width of the thermally damaged tissue was 687.22 ± 116.58 μm and 1157.51 ± 262.77 μm for TDFL and DL, respectively. A connective tissue capsule was formed at the rims of the wound. In individual animals, fat tissue from the mesentery partially filled the wound. On the border between the adhesions and spleen tissue, hemosiderin deposits were visible (Figure 5 and Figure 6, Table 2).

**Figure 7:**
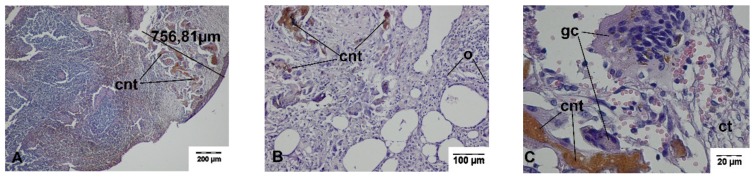
Histopathological sections of the spleen 14 days following cutting with the thulium-doped fiber laser (TDFL) (HE-staining). Black lines indicate the width of the entire zone of thermal damage. (**A**) A narrow streak of the residues of carbonized tissue in the cortex of the spleen (magnification × 40); (**B**) site of omentum adhesion to the spleen (magnification × 100); (**C**) multinucleated giant cells adjacent to the residues of carbonized tissue (magnification × 400). cnt, carbonized necrotic tissue; o, omentum; gc, giant cells; ct, connective tissue.

**Figure 8:**
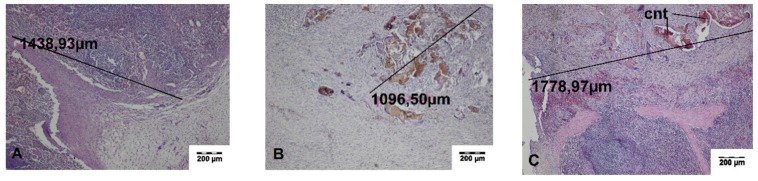
Histopathological sections of the spleen on day 14 after cutting with the diode laser (DL) (HE staining). Black lines indicate the width of the entire zone of thermal damage. (**A**) Site of omentum adhesion to the wound, a band of the fibrous connective tissue penetrating the spleen parenchyma are visible (magnification × 40); (**B**,**C**) residues of carbonized tissue surrounded by the connective tissue with proliferating splenocytes (magnification × 40). cnt, carbonized necrotic tissue.

Connective tissue with residues of the carbonized necrotic tissues surrounded by inflammatory cells, including giant cells, was found during histopathological evaluation of the cutting sites of the spleen obtained on day 14 after surgery. In the adjacent spleen parenchyma, proliferation of the connective tissue and splenocytes, which gradually changed into normal spleen tissue, was seen. The wound was covered with a connective tissue capsule. The average width of the thermal damage zone was 761.64 ± 34.33 μm and 1609.82 ± 202.22 μm for TDFL and DL, respectively. If the omentum adhered to the cutting sites, loose connective tissue or adipose tissue transforming into vascularized adipose tissue was present. At the border, hemosiderin deposits in the spleen and the formation of the connective tissue capsule on the spleen surface was seen. Occasionally, streaks of fibrous connective tissue penetrated the parenchyma up to 1500 μm (Figure 7 and Figure 8, Table 2).

## 4. Discussion

The development of laser technologies is accompanied by constant attempts to apply them in medicine. The efficacy of cutting, intraoperative hemostasis, and the possibility to control the impact on healthy tissues during laser surgery are the subjects of numerous studies; however, promising experimental results have not been achieved in clinical practice. Several physical methods of cutting and coagulation based on laser technologies, such as CO_2_ or Nd: YAG lasers, are available in the surgery of parenchymal organs. The Nd: YAG laser technology, which gives good effects, has been used, among others, in pediatric surgery of parenchymal organs or bronchial surgery [13,24].

Hemostatic transection of larger vessels is possible with the Nd: YAG laser emitting light at a wavelength of 1064 nm when the output power is 15–25 W. In animal model studies, veins up to 5 mm in diameter and arteries up to 2 mm were cut with good hemostasis. Spleen and other parenchyma organs were transected without bleeding over the next 3 weeks [13]. Hemostatic efficacy and good effects in partial spleen resection have also been demonstrated in comparative studies of infrared coagulation and the Nd: YAG laser in a dog model. Various efficacies of these technologies were demonstrated for other organs. For some of the organs, no positive effect was shown, which confirmed preceding clinical observations [25]. In the studies on the effectiveness of the CO_2_ and Nd, YAG lasers in 86 patients with liver, spleen, or kidney injuries, laser technology significantly reduced mortality in closed traumatic liver injuries and enabled spleen preservation in 46% of patients. Laser coagulation proved to effectively stop the parenchymal bleeding, thus increasing the quality of surgical procedures [9].

However, according to other studies, the CO_2_ laser provided good hemostasis only in 63% of patients, while in the remaining individuals, no positive effects were seen [8].

In the experiments on partial spleen resection with the Nd: YAG laser in a pig model, intraoperative hemostasis was achieved at a laser continuous output power of 80 W. Post-mortem examinations revealed no signs of bleeding. The width of the laser-induced tissue damage at the site of cutting was approximately 3000 μm [26].

These data indicate that hemostasis in the spleen may be achieved with the CO_2_ laser and other lasers operating at shorter wavelengths; however, the extent of the thermal damage of the adjacent tissue is either not known or reported to be high [13,26].

We applied a novel thulium-doped fiber laser to spleen incision and partial resection. This laser emits light at a wavelength of 1940 nm, which is absorbed by water 1000 times more when compared with radiation emitted by Nd: YAG lasers, and therefore does not penetrate deep into tissues. Besides the TDFL, we also applied the DL, operating at a 1470 nm wavelength, which is absorbed by the water 40 times more than the light emitted by Nd: YAG lasers. During the experiment, we used the lasers (1940 nm and 1470 nm) with the output power as low as could be acceptable for spleen surgery while inducing hemostasis. Based on a series of laser cuttings, we experimentally determined that 21 W for the Tm fiber laser and 50 W for the diode laser were the optimal values. Our main intention was to show that medium-power lasers can be effective tools for spleen surgery with minimal destruction of surrounding tissue, and that it is not necessary to apply more powerful or much more expensive lasers. We successfully performed incision or partial resection of the spleen in a pig model using TDFL and DL with no or minimal intraoperative bleeding which disappeared within 5 min.

During the post-mortem examination, we observed the normal process of wound healing on days 7 and 14 following surgery for both lasers. No signs of bleeding or clots were seen in the abdominal cavity. The streak of necrotic changes on the spleen after incision with DL was wider than after cutting with TDFL. 

Histopathological examination of the spleen fragments collected during surgery revealed a superficial zone of carbonization and a deeper zone of thermally changed tissue sharply demarcated from the normal parenchyma. In the superficial zone, a minimal exudation was visible. Both superficial and total thermal change zones were significantly wider for DL compared to TDFL (313.40 μm for DL vs. 197.20 μm for TDFL, and 1413.57 μm for DL vs. 655.26 μm for TDFL, respectively). 

These statistically significant differences persisted throughout the experiment. After 7 days, the superficial zone was 405.92-μm-wide for DL and 166.23-μm-wide for TDFL. The total change zone was larger after using DL as well (1157.51 μm for DL and 68.77 μm for TDFL). After 14 days, the total thermal change zone was 1609.82 μm for DL vs. 761.65 μm for TDFL.

We observed thinner bands of thermal changes in the spleen parenchyma after cutting with the thulium-doped fiber laser than with the diode laser during macroscopic and histopathological examinations. The entire zone of thermal damage was significantly wider for the diode laser in the specimens collected intraoperatively and on days 7 and 14 post-surgery, although it was narrower compared to the reported effects seen on the spleen after using Nd: YAG and CO_2_ lasers.

Similar zones of soft tissue thermal destruction were found in bovines’ tongues after cutting with a diode laser operating at a wavelength of 1470 and output power of 4 W. The width of the superficial zone reached 570 μm and the deep zone of cell membrane alterations measured up to 750 µm [27]. The same authors tested lasers emitting different wavelengths of 810, 980, 1470, and 1950 nm with a constant output power of 4 W, and they demonstrated that longer wavelengths, i.e., 1470 nm and 1940 nm, provided the highest regularity of incisions [27]. 

In studies on the impact of different laser wavelengths on ablative and thermal damage in the intervertebral discs, a laser emitting a wavelength of 1470 nm produced a wider zone of thermal changes (2.23 ± 1.02 mm) compared with our results [12].

The histological features of spleen healing after laser cutting in our study are consistent with the results of other authors investigating the use of lasers in spleen surgery [13,28].

A comparative analysis of the microscopic morphology of the wounds after laser coagulation in various parenchymal organs, including the spleen, demonstrated that inflammatory and repair processes were characterized by a predominance of proliferation. Laser thermal changes were always spatially separated from the intact parenchyma. It has been stated that coagulative necrosis (not involving the coagulation system) inhibits the exudative component of inflammation at the sites of the spleen and other organs exposed to laser energy. Early response of macrophages stimulates the proliferation of fibroblasts and the formation of fine scar tissue within 14 days [28].

The histological picture observed in our studies and the above-described histology are the result of thermal changes induced in the tissue because of the local increase of temperature at the place of radiation beam operation. This produces semi-circularly forming bands of tissue changes induced centrally by pyrolysis, carbonization, and vaporization, and peripherally by further coagulation, irreversible hyperthermia, and reversible hyperthermia. 

The effects of laser radiation on the tissue are determined by the parameters of the radiation delivered, but also by the differing thermal properties of the tissues themselves. The absorption properties of the main biological absorbers, including melanin, hemoglobin, water, and proteins, differ and influence the penetration depth of the laser beam. This translates into the dynamization of the reparative and regenerative processes taking place in wounds and those observed in the histological image [29].

In a study comparing the wounds after cutting with a laser and a scalpel, a delay in laser wound inflammation was found in the early healing period. It involved the production of collagen, re-epithelialization, and tensile strength of the wound. The delay in the healing process was similar to that observed after electrocoagulation and in burn wounds. Later stages of laser wound healing, however, compensated for the process of epithelialization, and the changes led to smaller contractions of the laser scars compared to those created using the scalpel [30].

TDFL belongs to a new generation of continuously developing technology systems. The number of preclinical reports on the local impact on parenchymatous organs is still small, although continually increasing, since these devices may potentially find many applications in medicine and surgery, as predicted by their properties [31,32].

Previous results on the use of a thulium fiber laser operating at a wavelength of 1940 nm in patients (population of 187 persons) for endobronchial therapy are promising. The thulium laser was considered a safe and versatile method for the treatment of airway narrowing and stent obstruction caused by tissue ingrowth compared with the potential advantages of the Nd: YAG laser (1064 nm). A laser output power from 5 to 20 W was considered safe. However, according to the authors, further comparative studies are needed [16]. 

## 5. Conclusions

Based on the results of our study, we conclude that both of the tested lasers have the potential to be used in clinical practice for hemostasis and partial resection of the spleen. The histologically confirmed zones of thermal tissue changes in the spleen after TDFL were almost twice as narrow as after DL and more than three times narrower than the lesions produced by the Nd: YAG laser described by other authors. The TDFL laser seems to be an effective tool for precise surgical procedures on the spleen with a narrow and controlled zone of destruction of the adjacent tissue.

## Figures and Tables

**Table 1:** Parameters of the lasers used during the study.

Parameters	Thulium-Doped Fiber Laser	Diode Laser
Type of laser	Solid-state laser	Semiconductor laser
Wavelength of operation	1940 nm	1470 nm
Power (CW)	21 ± 1 W	50 ± 1 W
Diameter of laser probe	400 μm	400 μm
NA of laser probe	0.22	0.22
Power density at the laser probe output	16.7 kW	39.8 kW
Exposure time	laser probes were moved along the surface at a speed of 3.34 ± 0.38 mm/s in a continuous pass	laser probes were moved along the surface at a speed of 3.34 ± 0.38 mm/s in a continuous pass

CW—continuous wave; NA—numerical aperture.

**Table 2:** Dimensions of the thermal damage zones produced by cutting the spleen with a thulium-doped fiber laser (TDFL) and a diode laser (DL) on histopathological sections collected intraoperatively and on days 7 and 14 following surgery.

Day of Experiment	Thulium-Doped Fiber Laser (μm)	Diode Laser (μm)
**a**	**Superficial Zone**	
Day 0 (*n* = 6)	197.29 ± 35.99 ^a^	313.44 ± 20.89 ^b^
Day 7 (*n* = 6)	166.23 ± 41.99 ^a^	405.92 ± 140.22 ^b^
**b**	**Total Width of the Thermal Damage Zone**	
Day 0 (*n* = 6)	655.26 ± 107.70 ^A^	1413.37 ± 111.85 ^B^
Day 7 (*n* = 6)	687.22 ± 116.58 ^a^	1157.51 ± 262.77 ^b^
Day 14 (*n* = 6)	761.65 ± 34.33 ^A^	1609.82 ± 202.22 ^B^

Data are expressed as mean ± standard deviation for *n* animals. Significantly different values in a row are signified with different lowercase letters (*p* < 0.05) or uppercase letters (*p* < 0.001) in superscript.
