# Addendum: Żywicka, B., et al. Comparison of a 1940 nm Thulium-Doped Fiber Laser and a 1470 nm Diode Laser for Cutting Efficacy and Hemostasis in a Pig Model of Spleen Surgery. *Materials* 2020, *13*, 1167

**DOI:** 10.3390/ma14040966

**Published:** 2021-02-18

**Authors:** Bogusława Żywicka, Zbigniew Rybak, Maciej Janeczek, Albert Czerski, Jolanta Bujok, Maria Szymonowicz, Maciej Dobrzyński, Mariusz Korczyński, Jacek Świderski

**Affiliations:** 1 Department of Experimental Surgery and Biomaterial Research, Wroclaw Medical University, Bujwida 44, 50-368 Wroclaw, Poland; zbigniew.rybak@umed.wroc.pl (Z.R.); maria.szymonowicz@umed.wroc.pl (M.S.); 2 Department of Animal Physiology and Biostructure, Division of Anatomy, Wroclaw University of Environmental and Life Sciences, Kożuchowska 1, 51-631 Wroclaw, Poland; maciej.janeczek@upwr.edu.pl; 3 Department of Animal Physiology and Biostructure, Division of Animal Physiology, Wroclaw University of Environmental and Life Sciences, C.K. Norwida 31, 50-375 Wroclaw, Poland; albert.czerski@upwr.edu.pl (A.C.); jolanta.bujok@upwr.edu.pl (J.B.); 4 Department of Conservative Dentistry and Pedodontics, Wroclaw Medical University, Krakowska 26, 50-425 Wroclaw, Poland; maciej.dobrzynski@umed.wroc.pl; 5 Department of Environment Hygiene and Animal Welfare, Wroclaw University of Environmental and Life Sciences, Chełmońskiego 38c, 51-630 Wroclaw, Poland; mariusz.korczynski@upwr.edu.pl; 6 Institute of Optoelectronics, Military University of Technology, Kaliskiego 2, 00-908 Warsaw, Poland; jacek.swiderski@wat.edu.pl

The authors would like to add the following sentence to the “Funding” section of their article [1]:

“The APC was financed under the Leading Research Groups support project from the subsidy increased for the period 2020–2025 in the amount of 2% of the subsidy referred to Art. 387 (3) of the Law of 20 July 2018 on Higher Education and Science, obtained in 2019.”

The authors would like to apologize for any inconvenience caused to the readers by these changes.
